# Effect of clinical features on antiseizure medication doses in patients with newly diagnosed epilepsy

**DOI:** 10.3389/fneur.2023.1159339

**Published:** 2023-08-07

**Authors:** Hire Hersi, Jukka Peltola, Jani Raitanen, Jukka T. Saarinen

**Affiliations:** ^1^Department of Neurology, Vaasa Central Hospital, Vaasa, Finland; ^2^Faculty of Medicine and Health Technology, Tampere University, Tampere, Finland; ^3^Department of Neurology, Tampere University Hospital, Tampere, Finland; ^4^Special Services Unit, Faculty of Social Sciences (Health Sciences), UKK Institute for Health Promotion Research, Tampere University, Tampere, Finland

**Keywords:** antiseizure medication, newly diagnosed epilepsy, seizure freedom, treatment outcomes, oxcarbazepine, valproic acid, carbamazepine

## Abstract

**Objective:**

We evaluate the effect of distinct clinical features on anti-seizure medication (ASM) doses in seizure-free and not seizure-free patients aged ≥16 years with new-onset epilepsy.

**Materials and methods:**

This study included 459 patients with a validated diagnosis of epilepsy. The most prescribed ASMs were oxcarbazepine (OXC; *n* = 307), followed by valproic acid (VPA; *n* = 115), carbamazepine (CBZ; *n* = 81), and lamotrigine (LTG; *n* = 67). The seizure freedom rate with their first or subsequent ASM was 88.0%. A retrospective analysis of patient records was performed to determine any association between doses of ASMs and patient characteristics.

**Results:**

The median OXC dose in seizure-free patients aged >60 years was 600 mg compared to 900 mg in younger patients. When controlling for age but not in an unadjusted model, the median dose of OXC was lower (300 mg, *p* = 0.018) for seizure-free patients compared to non-seizure-free patients, and the median dose of OXC was also 300 mg lower among older patients aged >60 years (*p* < 0.001). The median OXC doses for men aged ≤60 years were 300 mg higher than for women aged >60 years (900 mg vs. 600 mg, *p* = 0.021). The median dose of VPA was 400 mg higher in men than in women (*p* < 0.001) and 400 mg higher in not seizure-free patients compared to seizure-free patients only when adjusting for sex (*p* < 0.001). Higher median doses for CBZ were registered with FAS compared with FBTCS (difference in median doses of 200 mg; *p* = 0.017).

**Conclusion:**

Significant OXC dose differences were detected between age groups, whereas VPA dosing was different in men and women. Moreover, CBZ doses were dependent on some seizure types. These data allow for the individualization of the initial target dosing based on key clinical characteristics.

## 1. Introduction

Several clinical features have been recognized as prognostic factors for achieving seizure freedom in patients with newly diagnosed epilepsy. The response to the first antiseizure medication (ASM) trial is of paramount importance, as most patients achieving seizure freedom respond to the initial adequate ASM trial defined by the International League Against Epilepsy guidelines (1). We recently identified favorable prognostic factors for seizure freedom on the first ASM trial, including male sex, unknown etiology, no epileptiform activity on electroencephalography (EEG), and presenting seizure types of focal to bilateral tonic-clonic seizures (FBTCS) or focal aware seizures (FAS) (2). Subsequently, we assessed the same prognostic factors on the second or following ASM trials in another study, which found that an age >60 years and absence of epileptiform activity on EEG were favorable prognostic features of seizure freedom (3). These features associated with a favorable prognosis of seizure freedom may also be of importance in determining appropriate doses of a given ASM to achieve seizure freedom, an aspect seldom explored.

There is a changing landscape regarding the age distribution of new-onset epilepsy, emphasizing the need for more granular knowledge of ASM responses based on different age categories. Throughout the last four decades in Finland, the incidence of epilepsy in individuals aged <65 years has been mostly stable, but there has been a nearly 5-fold increase in the rate of new-onset epilepsy in the older population (4). Changes in the prevalence of epilepsy across age groups have important implications for the choice of ASM because different age groups may exhibit different responses to therapy and dose-response characteristics (5). Older patients have a higher serum ASM concentration than younger adults for the same administered dose; thus, the ASM dose must be carefully selected when treating older patients (6). However, no formal clinical practice guidelines exist for patients aged >60 years with new-onset epilepsy (7).

Furthermore, there is emerging data regarding the effect of other clinical features on ASM doses achieving favorable clinical outcomes. In a recent study, the mean doses of most ASMs used in monotherapy were lower in women than in men among adult patients with epilepsy (8). However, there is a paucity of clinical data regarding the effect of sex on the dosing of different ASMs. Moreover, there is even a larger gap in knowledge on the effect of etiology, seizure types, or EEG characteristics on ASM dosing in patients responding to ASM therapy with seizure freedom (9).

This study aimed to evaluate the ASM dosing both in seizure-free and not seizure-free patients aged ≥16 years with newly diagnosed epilepsy based on clinical features, including age, sex, presenting seizure type, etiology, and EEG findings.

## 2. Materials and methods

The population sampled comprised 584 patients aged ≥16 years who were referred to the Tampere University Hospital between 1 January 1995 and 31 December 2005, following a diagnosis of new-onset epilepsy. All patients were retrospectively followed up until 31 December 2006 until 1 year of seizure freedom was achieved, or until patient death. The medical records of patients, including clinic visits and clinical information, were retrospectively examined, and the demographic information of all patients was collected. Neuroimaging, particularly computed tomography or magnetic resonance imaging, was performed and evaluated by neuroradiologists to screen for underlying structural abnormalities. The evaluation of etiology was based mainly on imaging results and clinical history. The detailed clinical characteristics of all 459 patients with validated newly diagnosed epilepsy who were included in this study cohort are presented in our previous studies (2, 3).

For all patients diagnosed with epilepsy, ASM therapy was initiated according to local standard clinical practice at the time of data collection. Patients were followed up in the epilepsy clinic according to routine clinical practice. At the follow-up visit, clinical information and responses to ASM therapy (either monotherapy or polytherapy) were recorded. The ASM doses were adjusted according to efficacy and tolerability at the discretion of the treating physician.

Data were analyzed using Stata version 17.0 for Windows (College Station, TX: StataCorp LLC). Doses are described as medians with interquartile ranges. We examined the relationships between seizure freedom (patients who did not become seizure-free as a reference group) and doses using a bootstrapped median regression model. Unadjusted models (column “Model 1” in **Table 2**) and models adjusted for age at the date of diagnosis, sex, seizure type, EEG, and etiology (Model 2 in **Table 2**, all models in **Tables 3**–**7**) were constructed. We used 200 bootstrap replications to obtain an estimate of the variance-covariance matrix of the estimators (standard errors).

We also examined the joint effect of age at the date of diagnosis and sex on the dose of oxcarbazepine (OXC), valproic acid (VPA), and carbamazepine (CBZ) by fitting a bootstrapped median regression model. The joint variable was categorized into four groups: 1 = male ≤60 years (reference group), 2 = female >60 years, 3 = male >60 years, and 4 = female ≤60 years.

In this retrospective study, there was no contact with patients, and information was collected from the patient register of the Tampere University Hospital. This study does not require ethics committee approval, according to the Finnish Law on Research. Following Finnish guidelines, this study was approved by the head of the Tampere University Science Center.

## 3. Results

The study cohort comprised 251 (54.7%) male patients and 208 (45.3%) female patients. The median age at diagnosis was 45 years; 76.9% (353/459) of patients were aged ≤60 years, whereas 23.1% (106/459) were aged >60 years. The seizure freedom rate with the first or subsequent ASM was 88.0% (404/459). A total of 308 patients (75.9% of all patients achieving seizure freedom) became seizure-free following the administration of the first ASM regimen; 59 of 151 patients (14.5% of all patients achieving seizure freedom) became seizure-free following the administration of the second ASM regimen; and 37 patients became seizure-free after the third to fifth ASM regimens when all ASM trials were counted. The most prescribed ASMs were OXC (307, 66.9%), followed by VPA (115 25.1%), CBZ (81, 17.6%), and lamotrigine (LTG, 67 [14.5%]) (Table 1). Among the patients who achieved 1-year seizure freedom in the entire cohort, 10.1% (41/404) were on combination therapy.

**Table 1:** Clinical features categorized based on ASM response.

	**All patients**	**Seizure-free after the first ASM trial**	**Seizure-free after the second or subsequent ASM regimens**	**Persistent seizures**
*n*	459	308	96	55
**Sex*****, n*** **(%)**				
Female	208 (45.3)	125 (40.6)	54 (56.2)	29 (52.7)
Male	251 (54.7)	183 (59.4)	42 (43.8)	26 (47.3)
**Age at the date of diagnosis*****, n*** **(%)**				
≤ 60 years	353 (76.9)	237 (76.9)	69 (71.9)	47 (85.5)
>60 years	106 (23.1)	71 (23.1)	27 (28.1)	8 (14.5)
**Etiology*****, n*** **(%)**				
**Structural**				
Benign tumor	19 (4.1)	10 (3.2)	6 (6.3)	3 (5.5)
Hippocampal sclerosis	3 (0.7)	1 (0.3)	2 (2.1)	0
Malformation of cortical development	11 (2.4)	5 (1.6)	3 (3.1)	3 (5.5)
Malignant tumor	21 (4.6)	10 (3.2)	6 (6.3)	5 (9.1)
Other hippocampal pathology	12 (2.6)	8 (2.6)	1 (1.0)	3 (5.5)
Perinatal injury	5 (1.1)	4 (1.3)	1 (1.0)	0
Traumatic brain injury	27 (5.9)	19 (6.2)	3 (3.1)	5 (9.1)
Vascular lesion	113 (24.6)	69 (22.4)	27 (28.1)	17 (30.9)
Vascular malformation	30 (6.5)	21 (6.8)	7 (7.3)	2 (3.6)
Genetic	25 (5.4)	18 (5.8)	7 (7.3)	0
Infectious	15 (3.3)	9 (2.9)	3 (3.1)	3 (5.5)
Unknown	178 (38.8)	134 (43.5)	30 (31.3)	14 (25.5)
**Epilepsy type*****, n*** **(%)**				
Focal	434 (94.6)	290 (94.2)	89 (92.7)	55 (100)
Generalized	25 (5.4)	18 (5.8)	7 (7.3)	0
**Type of first seizure*****, n*** **(%)**				
FBTCS	320 (69.7)	222 (72.1)	63 (65.6)	35 (63.6)
FAS	75 (16.3)	50 (16.2)	15 (15.6)	10 (18.2)
FIAS	40 (8.7)	19 (6.2)	11 (11.5)	10 (18.2)
GTCS	15 (3.3)	12 (3.9)	3 (3.1)	0
Myoclonic	9 (2.0)	5 (1.6)	4 (4.2)	0
**EEG*****, n*** **(%)**				
Normal	188 (41.0)	137 (44.5)	36 (37.5)	15 (27.3)
Epileptiform activity	103 (22.4)	61 (19.8)	26 (27.1)	16 (29.1)
Focal slowing	66 (14.4)	46 (14.9)	12 (12.5)	8 (14.5)
Unspecific	52 (11.3)	34 (11.0)	7 (7.3)	11 (20.0)
No EEG	50 (10.9)	30 (9.7)	15 (15.6)	5 (9.1)
**ASM**				
Benzodiazepine*, n* (%)	2 (0.4)	0	0	2 (3.6)
Carbamazepine*, n* (%)	81 (17.6)	54 (17.5)	18 (18.8)	9 (16.4)
Clobazam*, n* (%)	11 (2.4)	0	4 (4.2)	7 (12.7)
Clonazepam*, n* (%)	8 (1.7)	1 (0.3)	4 (4.2)	3 (5.5)
Gabapentin*, n* (%)	19 (4.1)	1 (0.3)	9 (9.4)	9 (16.4)
Lamotrigine*, n* (%)	67 (14.6)	12 (3.9)	35 (36.5)	20 (36.4)
Levetiracetam*, n* (%)	43 (9.4)	4 (1.3)	22 (22.9)	17 (30.9)
Oxcarbazepine*, n* (%)	307 (66.9)	184 (59.7)	74 (77.1)	49 (89.1)
Phenobarbital*, n* (%)	1 (0.2)	0	0	1 (1.8)
Phenytoin*, n* (%)	27 (5.9)	8 (2.6)	15 (15.6)	4 (7.3)
Pregabalin*, n* (%)	2 (0.4)	0	0	2 (3.6)
Tiagabine*, n* (%)	14 (3.1)	0	8 (8.3)	6 (10.9)
Topiramate*, n* (%)	31 (6.8)	2 (6.5)	15 (15.6)	14 (25.5)
Valproic acid*, n* (%)	115 (25.1)	51 (16.6)	47 (49.0)	17 (30.9)

FBTCS, focal to bilateral tonic-clonic seizures; FAS, focal aware seizures; FIAS, focal impaired awareness seizures; GTCS, generalized tonic-clonic seizures; EEG, electroencephalography; ASM, anti-seizure medication.

Based on the unadjusted median regression model, no differences in median doses of OXC or other ASMs between seizure-free and not seizure-free patients with focal epilepsy receiving mono- or polytherapy were found (Table 2, Model 1). However, after controlling for age, the median dose of OXC was significantly lower (300 mg, *p* = 0.018) for seizure-free patients compared to not seizure-free patients (Table 2, Model 2). Moreover, there was also a significant relationship between age and dose of OXC; the median dose of OXC was 300 mg lower among older patients aged >60 years than among their younger counterparts (*p* < 0.001). Finally, the median OXC dose in seizure-free patients aged >60 years was 600 mg compared to 900 mg in younger patients. There were no significant differences in other ASMs.

**Table 2:** Antiseizure medication doses (median and interquartile range) in either mono- or polytherapy in categories of age in patients with focal epilepsy.

		**Age** ≤ **60 years**		**Age** >**60 years**		**Model 1**	**Model 2**	
	**szf**	***n***	**Median (IQR)**	***n***	**Median (IQR)**	**Est. (p)** ^a^	**Est. (p)** ^b^	**Est. (p)** ^c^
Oxcarbazepine	Yes	178	900 (600)	34	600 (300)	0 (1.000)	−300 (0.018)	−300 (< 0.001)
	No	71	1,200 (900)	22	600 (300)		
Carbamazepine	Yes	43	400 (200)	15	400 (200)	−200 (0.058)	−200 (0.070)	0 (1.000)
	No	17	750 (400)	5	400 (200)		
Valproic acid	Yes	34	1,000 (400)	30	900 (400)	0 (1.000)	−100 (0.616)	−100 (0.483)
	No	20	1,100 (950)	10	1,000 (800)		
Lamotrigine	Yes	27	200 (300)	2	138 (125)	−100 (0.075)	−100 (0.161)	0 (1.000)
	No	29	300 (200)	3	300 (400)		
Phenytoin	Yes	3	200 (300)	13	250 (100)	−50 (0.382)	−50 (0.555)	0 (1.000)
	No	5	300 (100)	6	250 (200)		
Levetiracetam	Yes	14	1,000 (1.000)	3	1,000 (0)	−500 (0.278)	−500 (0.299)	0 (1.000)
	No	19	1,500 (1,500)	3	1,000 (700)		
Topiramate	Yes	8	300 (225)	2	175 (150)	0 (1.000)	50 (0.635)	−50 (0.631)
	No	18	275 (200)	1	200		
Gabapentin	Yes	4	2,600 (800)	3	900 (400)	200 (0.770)	400 (0.397)	−1,200 (0.026)
	No	10	1,900 (800)	2	1,500 (600)			

Other antiseizure medications were used in less than five patients.

szf, seizure freedom; IQR, interquartile range; n, number; Coef., estimate from the median regression model; p, significance; na, convergence not achieved.

Model 1: outcome = antiseizure medication dose, exposure variable = seizure freedom.

Model 2: outcome = antiseizure medication dose, exposure variables = seizure freedom and age.

a estimate and significance of the seizure freedom (ref. = no) from model 1.

b estimate and significance of the seizure freedom (ref. = no) from model 2.

c estimate and significance of the age (ref. = ≤ 60) from model 2.

In a similar analysis adjusted for sex, no difference was found in median doses of OXC between men and women or between seizure-free and not seizure-free patients (Model 1 in Table 3). However, when adding age to the model, a significant relationship between OXC dose and seizure-free and not seizure-free patients was found (the median dose was 300 mg less in seizure-free patients; *p* = 0.032). Conversely, for VPA using the same model, there was a significant relationship: the median dose of VPA was 400 mg higher in men than in women (*p* < 0.001) and, also, 400 mg higher in not seizure-free patients compared to seizure-free patients (*p* < 0.001). The latter finding remained when adding age as an adjustment (Model 2 in Table 3). The median VPA dose in seizure-free women was 750 mg, compared to 1,000 mg in men. There were no significant differences in medians between sexes in other ASMs (Table 3).

**Table 3:** Antiseizure medication doses (median and interquartile range) in either mono- or polytherapy according to sex in patients with focal epilepsy.

		**Women**		**Men**		**Model 1**		**Model 2**
	**szf**	***n***	**Median (IQR)**	***n***	**Median (IQR)**	**Est. (p)** ^a^	**Est. (p)** ^b^	**Est. (p)** ^c^
Oxcarbazepine	Yes	90	900 (600)	122	900 (300)	0 (1.000)	0 (1.000)	−300 (0.032)
	No	51	900 (750)	42	1,425 (900)			
Carbamazepine	Yes	25	400 (200)	33	400 (200)	−200 (0.065)	0 (1.000)	−200 (0.057)
	No	12	600 (400)	10	650 (400)			
Valproic acid	Yes	22	750 (400)	42	1,000 (400)	−400 (< 0.001)	400 (< 0.001)	−400 (0.002)
	No	18	950 (400)	12	1,800 (500)			
Lamotrigine	Yes	17	200 (200)	12	150 (200)	−100 (0.188)	0 (1.000)	−100 (0.174)
	No	19	200 (350)	13	350 (100)			
Phenytoin	Yes	10	225 (100)	6	250 (100)	−100 (0.111)	−50 (0.420)	−50 (0.528)
	No	6	350 (150)	5	300 (100)			
Levetiracetam	Yes	8	1,000 (1,500)	9	1,000 (0)	−500 (0.201)	0 (1.000)	−500 (0.253)
	No	11	2,000 (1,500)	11	1,000 (1,500)			
Topiramate	Yes	2	250 (300)	8	225 (225)	50 (0.583)	−100 (0.229)	100 (0.294)
	No	9	300 (200)	10	200 (250)			
Gabapentin	Yes	4	1,450 (1,750)	3	2,400 (1,600)	400 (0.520)	800 (0.071)	400 (0.349)
	No	5	1,200 (4,009)	7	2,000 (600)			

Other antiseizure medications were used in less than five patients.

szf, seizure freedom; IQR, interquartile range; n, number; Est., estimate from the median regression model; p, significance.

Model 1: outcome = antiseizure medication dose, exposure variables = seizure freedom and sex.

Model 2: outcome = antiseizure medication dose, exposure variables = seizure freedom, sex, and age.

a estimate and significance of the seizure freedom (ref. = no) from model 1.

b estimate and significance of the sex (ref. = female) from model 1.

c estimate and significance of the seizure freedom (ref. = no) from model 2.

When combining age and sex categories into four different combinations (male ≤60 years, male >60 years, female ≤60 years, female >60 years), the median OXC doses for men aged ≤60 years was 300 mg higher than for women >60 years (900 mg vs. 600 mg, *p* = 0.021). When applying the same analysis for VPA, significant findings emerged with women in both age groups with 400 mg lower median doses compared with men ≤60 years (600 mg vs. 1,000 mg).

There were no significant differences in the doses of OXC according to seizure type (Table 4). In Model 2, where the outcome was defined as ASM dose and exposure variables were seizure freedom and seizure type, significantly higher median doses for CBZ were registered with FAS compared with FBTCS (difference in median doses of 200 mg; *p* = 0.017).

**Table 4:** Antiseizure medication doses (median and interquartile range) in either mono- or polytherapy according to seizure type in patients with focal epilepsy.

		**Seizure-free**		**Not seizure-free**		**Model**		
	**sz type**	***n***	**Median (IQR)**	***n***	**Median (IQR)**	**Est. (p)** ^a^	**Est. (p)** ^b^	**Est. (p)** ^c^
Oxcarbazepine	FBTCS	157	900 (600)	63	900 (900)	0 (1.000)	0 (1.000)	300 (0.115)
	FAS	40	900 (600)	18	900 (600)			
	FIAS	15	1,200 (600)	12	1,275 (900)			
Carbamazepine	FBTCS	47	400 (200)	13	600 (400)	−200 (0.082)	200 (0.017)	0 (1.000)
	FAS	9	600 (100)	5	600 (400)			
	FIAS	2	400 (0)	4	725 (125)			
Valproic acid	FBTCS	48	900 (400)	20	1,000 (550)	−100 (0.576)	100 (0.656)	100 (0.718)
	FAS	10	1,000 (600)	4	1,200 (1,450)			
	FIAS	6	800 (400)	6	1,500 (600)			
Lamotrigine	FBTCS	21	200 (200)	21	300 (300)	−100 (0.074)	200 (0.248)	0 (1.00)
	FAS	4	500 (200)	4	200 (150)			
	FIAS	4	175 (175)	7	400 (300)			
Phenytoin	FBTCS	14	200 (100)	7	250 (200)	−50 (0.385)	100 (0.103)	100 (0.116)
	FAS	1	300	2	350 (100)			
	FIAS	1	300	2	350 (100)			
Levetiracetam	FBTCS	11	1,000 (1,000)	16	1,000 (750)	0 (1.000)	2,000 (0.090)	1,000 (0.138)
	FAS	1	3,000	1	1,000			
	FIAS	5	1,000 (0)	5	3,000 (500)			
Topiramate	FBTCS	7	250 (250)	10	250 (250)	−50 (0.583)	−50 (0.572)	0 (1.000)
	FAS	1	200	3	250 (100)			
	FIAS	2	250 (300)	6	250 (200)			
Gabapentin	FBTCS	4	2,400 (1,550)	7	1,800 (800)	0 (1.000)	−800 (0.063)	400 (0.342)
	FAS	2	1,000 (400)	2	1,000 (400)			
	FIAS	1	2,400	3	2,400 (1,000)			

Other antiseizure medications were used in fewer than five patients.

szf, seizure freedom; IQR, interquartile range; n, number; Est., estimate from the median regression model; p, significance.

Model: outcome = antiseizure medication dose, exposure variables = seizure freedom and first seizure type.

a estimate and significance of the seizure freedom (ref. = no).

b estimate and significance of the first seizure type (FAS vs. FBTCS).

c estimate and significance of the first seizure type (FIAS vs. FBTCS).

The CBZ median dose was 200 mg higher among patients with unspecific activity in EEG compared to patients with normal EEG (*p* = 0.031). In contrast, the median VPA dose was 400 mg lower in patients with unspecific activity in EEG compared to patients with normal EEG (*p* = 0.006). The median doses of other ASMs with focal epilepsy were not significantly influenced by EEG characteristics (Table 5). The median doses of ASMs were not influenced by etiology (Table 6).

**Table 5:** Antiseizure medication doses (median and interquartile range) in either mono- or polytherapy in categories of EEG in patients with focal epilepsy.

		**Normal**		**Epileptiform activity**			**Unspecific changes**	
	**szf**	***n***	**Median (IQR)**	***n***		**Median (IQR)**	***n***	**Median (IQR)**
Oxcarbazepine	Yes	95	900 (600)	42		900 (600)	21	900 (450)
	No	29	900 (1,200)	24		975 (900)	15	1,200 (900)
Carbamazepine	Yes	30	400 (200)	9		600 (400)	4	600 (100)
	No	7	800 (200)	8		400 (250)	2	900 (300)
Valproic acid	Yes	26	1,000 (400)	15		900 (400)	8	600 (750)
	No	10	1,250 (800)	9		1,500 (800)	3	1,000 (400)
Lamotrigine	Yes	15	200 (300)	5		200 (0)	4	175 (75)
	No	12	250 (325)	10		400 (200)	8	200 (150)
Phenytoin	Yes	5	200 (50)	1		200	2	500 (600)
	No	1	400	8		275 (200)	0	
Levetiracetam	Yes	1	3,000	8		1,000 (1,000)	2	1,000
	No	8	1,000 (250)	7		2,000 (2,000)	4	1,500 (1,000)
Topiramate	Yes	4	225 (125)	2		250 (300)	1	150
	No	6	275 (200)	8		200 (200)	2	400 (0)
Gabapentin	Yes	2	1,600 (800)	0			0	
	No	2	2,600 (400)	3	1,933 (416)	1,800 (800)	1	1,800
					**Focal slowing**		**No EEG**	
					***n***	**Median (IQR)**	***n***	**Median (IQR)**
Oxcarbazepine	Yes			34		900 (600)	20	825 (450)
	No			15		1,200 (600)	10	750 (1,200)
Carbamazepine	Yes			7		400 (200)	8	450 (200)
	No			2		500 (200)	3	800 (800)
Valproic acid	Yes			7		1,000 (600)	8	1,000 (500)
	No			4		750 (900)	4	600 (550)
Lamotrigine	Yes			2		350 (300)	3	200 (425)
	No			1		100	1	200
Phenytoin	Yes			4		250 (100)	4	275 (75)
	No			1		300	1	300
Levetiracetam	Yes			4		1,000 (750)	2	1,500 (1,000)
	No			3		3,000 (2,000)	0	
Topiramate	Yes			3		400 (300)	0	
	No			1		200	0	
Gabapentin	Yes			3		2,400 (1,900)	2	2,000 (2,400)
	No			3		1,800 (800)	3	1,200 (1,600)

Other antiseizure medications were used in less than five patients.

szf, seizure freedom; IQR, interquartile range; n, number.

**Table 6:** Antiseizure medication doses (median and interquartile range) in either mono- or polytherapy according to etiology in patients with focal epilepsy.

		**Structural/Infectious**		**Unknown**		**Model**	
	**szf**	***n***	**Median (IQR)**	***n***	**Median (IQR)**	**Est. (p)** ^a^	**Est. (p)** ^b^
Oxcarbazepine	Yes	116	900 (600)	96	900 (600)	0 (1.000)	0 (1.000)
	No	66	900 (1,200)	27	900 (900)		
Carbamazepine	Yes	36	400 (200)	22	400 (200)	−200 (0.068)	0 (1.000)
	No	14	650 (400)	8	600 (400)		
Valproic acid	Yes	35	1,000 (600)	29	900 (400)	−100 (0.542)	100 (0.383)
	No	22	1,300 (900)	8	1,000 (250)		
Lamotrigine	Yes	14	200 (300)	15	200 (300)	−100 (0.094)	0 (1.000)
	No	19	200 (200)	13	300 (300)		
Phenytoin	Yes	13	200 (100)	3	250 (100)	−100 (0.064)	−100 (0.055)
	No	9	300 (100)	2	400 (0)		
Levetiracetam	Yes	12	1,000 (1,000)	5	1,000 (0)	−500 (0.290)	0 (1.000)
	No	14	1,250 (1,500)	8	1,250 (1,250)		
Topiramate	Yes	8	300 (275)	2	225 (50)	0 (1.000)	50 (0.605)
	No	12	325 (225)	7	200 (100)		
Gabapentin	Yes	6	1,800 (1,900)	1	2,000	600 (0.458)	400 (0.609)
	No	12	1,800 (1,000)	0			

Other antiseizure medications are used in less than five patients.

szf, seizure freedom; IQR, interquartile range; n, number; Est., estimate from the median regression model; p, significance.

Model: outcome = antiseizure medication dose, exposure variables = seizure freedom and etiology.

a estimate and significance of the seizure freedom (ref. = no).

b estimate and significance of the etiology (ref. = unknown).

Among all patients with focal epilepsy using OXC in monotherapy on either the first ASM regimen or first substitution, in an estimate comparing OXC doses between seizure-free and not seizure-free patients applying five models separately adjusted for sex, age, seizure type, EEG, and etiology, no significant findings emerged (Table 7).

**Table 7:** Oxcarbazepine doses according to clinical features (median and interquartile range) in monotherapy as either the first antiseizure medication or first substitution in patients with focal epilepsy.

	**Seizure-free**		**Not seizure-free**		
	**n**	**Median (IQR)**	***n***	**Median (IQR)**	**Est. (p)** ^a^
Sex					0 (1.000)
Male	114	900 (300)	22	900 (600)	
Female	79	900 (300)	37	750 (300)	
Age of diagnosis					0 (1.000)
≤ 60 years	161	900 (600)	40	900 (600)	
>60 years	32	600 (300)	19	600 (300)	
Seizure type					0 (1.000)
FBTCS	142	900 (450)	42	600 (300)	
FAS	39	900 (600)	13	900 (0)	
FIAS	12	900 (450)	4	750 (300)	
EEG					0 (1.000)
Normal	88	900 (450)	21	900 (300)	
Epileptiform activity	35	900 (600)	14	675 (300)	
Unspecific activity	21	900 (450)	8	750 (600)	
Focal slowing	32	900 (600)	9	900 (300)	
Etiology					0 (1.000)
Structural	98	900 (600)	39	900 (300)	
Infectious	5	1,500 (750)	2	900 (600)	
Unknown	90	900 (600)	18	900 (300)	

IQR, interquartile range; FBTCS, focal to bilateral tonic-clonic seizures; FAS, focal aware seizures; FIAS, focal impaired awareness seizures; EEG, electroencephalography.

a estimate and significance of the seizure freedom (ref. = no), five models separately adjusted for sex, age, seizure type, EEG, and etiology.

In the 17 patients with generalized epilepsy who achieved seizure freedom, the median dose of VPA was 900 mg in monotherapy or polytherapy. The doses of VPA were not significantly different between women and men (950 mg vs. 900 mg).

## 4. Discussion

The present study provides new insights into the median ASM doses based on clinical features and patient characteristics. Due to the distribution of ASM in our study, we were able to provide meaningful analysis results mainly for OXC, VPA, and CBZ. Significant OXC dose differences were detected between age groups, whereas VPA dosing was different in men and women. Moreover, CBZ doses were dependent on some seizure types and EEG findings.

Age was the main factor influencing ASM doses in this study. Patients aged ≤60 years needed higher doses of OXC to achieve seizure freedom compared with older patients, whereas the doses of other ASMs showed no statistically significant differences. However, in our study, the median dose of VPA for patients achieving seizure freedom was 1,000 mg for those younger than 60 years and 900 mg for those over 60 years. This finding is in line with a previously published study showing that 83.3% of older patients became seizure-free with a mean VPA monotherapy dose of 626 mg (10). In contrast, in a cohort of younger (mean age of 33 years) focal epilepsy patients with a seizure freedom rate of 62%, an average dose of 1,066 mg of VPA was required (11).

In our previous studies examining the percentage of seizure-free patients, age did not have an effect on patients achieving seizure freedom on their first ASM regimen (2), whereas in patients with failed achievement of seizure freedom on their first ASM regimen, an age >60 years was a favorable prognostic factor for seizure freedom with subsequent ASM regimens (3). However, it is unclear if decisions to modify doses were based only on the achievement of seizure freedom or on caution over age-related exposure and adverse events with ASMs in this study. The median dose of OXC (600 mg) was slightly lower in patients >60 years with focal epilepsy than in a previously published older cohort (874 mg) (12). In another study, seizure freedom was achieved with a lower mean daily dose of OXC in older individuals (900 mg/day) compared to mean doses of 1,200 mg/day in the whole cohort (13). Similarly, in our study, the median difference in OXC dose was 300 mg between older women and younger men. Age-related differences can partly be explained by drug disposition and elimination. A comparative pharmacokinetic study of OXC in older (age, 60–82 years) vs. young (age, 18–32 years) healthy volunteers showed that the mean concentrations of the OXC metabolite (monohydroxy derivative) were higher in the former population than in the latter population (14). OXC has been shown to have a good safety profile among older patients, which is consistent with the safety outcomes and adverse event rates noted in the general population. However, the concomitant use of drugs in this age group (polypharmacy) and pre-existing chronic conditions and comorbidities may influence drug safety and adverse event rates (15). The rate of hyponatremia linked to OXC therapy is higher in older patients, which may be an important consideration for dose adjustment and therapeutic monitoring (16).

Epilepsy management in older patients should consider a range of factors linked to the efficacy and safety outcomes of ASMs in this context (17), including diagnosed epileptic syndrome, patient sex, comorbidities, concomitant medications, tolerability, and safety of ASM, while ensuring compatibility with local guidelines. Similarly, the decision to increase or decrease the doses of ASM should be guided by these factors to reflect an individualized assessment process (18, 19), which also denotes that patient age also plays a significant role in influencing health outcomes during the ASM treatment process (20, 21).

In our previous publication, addressing the effect of sex on seizure outcomes in the same patient population, men with focal epilepsy were more likely to achieve seizure freedom than their female counterparts on their first ASM (2) but not with subsequent ASM regimens (3). In a recent Taiwanese study not taking seizure outcomes into consideration, the mean doses of OXC in monotherapy were 641 and 614 mg for men and women, respectively (*p* = 0.024). The authors speculated that this could be attributable to the fact that men typically weigh more than women (8). There is no clear evidence that sex differences influence the efficacy of OXC therapy among patients with focal epilepsy (22). Therefore, the reasons underlying this observation are unclear, potentially reflecting differences in patient risk profiles or other factors that were not investigated in this study. While the adjustment of OXC dose may be based on weight in children (23), there is no evidence that weight needs to be considered in adults, excluding the possibility of anthropometric differences between male and female patients accounting for variations in OXC dosing (24). OXC is known to be a weak inducer of CYP3A4, which plays a role in estrogen metabolism and may reduce the efficacy of oral contraceptive pills if used at high doses (24). However, it is unlikely that prescribers avoided higher doses of OXC to avoid drug-drug interactions in women taking oral contraceptive pills since the mean dose of OXC was higher among women who did not become seizure-free than among those who did. Seizure freedom may be achieved with a lower dose of OXC in women with focal epilepsy. In our study, female patients with focal epilepsy required lower doses of VPA to achieve seizure freedom. This is in line with a previous finding that the mean doses of VPA in monotherapy were 1,139 mg and 969 mg for men and women, respectively (*p* < 0.001) (8). Further studies to explore the influence of sex on ASM prescription in this population are warranted.

It was also noted that the CBZ median dose was significantly higher with FAS compared with FBTCS, which is consistent with our previous finding that patients with FBTCS as the presenting seizure type are more likely to achieve seizure freedom (2). There is no evidence in the literature to suggest the variable efficacy of ASM for achieving seizure freedom in different seizure types in patients with focal epilepsy (25). However, further research to confirm the potential for variation, as noted in this study, and explore the implications of drug dose optimizations is warranted. In the present study, some significant differences in median doses between patients with normal and unspecific EEG findings were detected; however, there were no statistically significant differences in seizure freedom rate, and these findings are difficult to interpret in a clinical context. There were no differences in ASM dosing regarding etiology.

This study has some limitations. First, the data were collected from a distinct geographical region and were retrospective in nature, potentially limiting the generalizability and reliability of the dataset. Furthermore, the analysis of patients who achieved seizure freedom on ASM therapy was further restricted by a lack of available data on all concomitant medications, making possible drug-drug interactions unknown. Additionally, we did not have information about comorbidities, adherence to medication, or lifestyle factors that may have influenced dose decisions. We did not analyze separately whether a given ASM was discontinued due to a lack of efficacy or due to tolerability issues for each subanalysis to maintain conciseness and clarity.

The analysis of ASM doses in patients with generalized epilepsy was limited by the small sample size and decreasing statistical power for the key outcomes of the study. The lack of serum level measurements is also a limitation of our study methodology. However, according to Finnish guidelines, ASM concentration measurements have not been routinely recommended due to their large intra- and interindividual variability and undetermined clinical significance, especially for OXC (26).

Finally, our cohort comprised patients initiating their ASM treatment between 1995 and 2005 prior to the widespread use or availability of newer ASMs, including only a restricted number of patients using LTG or LEV. However, due to the reimbursement policy, CBZ, OXC, or VPA are currently chosen as first-line ASMs for focal epilepsy in Finland (26) and are still widely used globally (8).

There have also been developments in the fields of neuroimaging and neurophysiology, but their effect has been greater for the management of drug-resistant epilepsy than for the initial diagnosis. Furthermore, all epilepsy diagnoses were re-evaluated using the new criteria for the definition of epilepsy (2). Owing to our study design, an initial seizure freedom rate of at least 1 year was used; however, long-term seizure freedom rates should be evaluated in the future to enhance the clinical relevance of these findings.

This study demonstrated that the doses of ASMs associated with seizure freedom in patients with epilepsy were influenced by age for OXC and by sex for VPA. The largest dose differences were observed between men aged ≤60 years and women aged >60 years. Significant dose differences were inconsistent across different ASMs, and further research is needed to clarify the effects of age and sex on ASM efficacy and prescription practices due to limitations inherent to the retrospective design of our study.

## Data availability statement

The original contributions presented in the study are included in the article/supplementary material, further inquiries can be directed to the corresponding author.

## Ethics statement

Ethical review and approval was not required for the study on human participants in accordance with the local legislation and institutional requirements. Written informed consent from the participants' legal guardian/next of kin was not required to participate in this study in accordance with the national legislation and the institutional requirements.

## Author contributions

JP and JS conceived and designed the study plan. JS and HH drafted the manuscript. JR performed the statistical analyses and aided in manuscript writing. All authors read and reviewed the manuscript and approved the final version.
